# SIRT1 deacetylates PKM2 to constrain lactate production and protect against premature ovarian insufficiency

**DOI:** 10.1093/lifemedi/lnag022

**Published:** 2026-06-09

**Authors:** Xiangrong Cui, Chongyang Han, Puhua Zhang, Ruotong Ju, Ruixiang Zhu, Jiali Luo, Li Peng, Chenyu Jia, Xinyu Zhu, Shu Wang, Huihui Li, Tingting Xue, Meng Zou, Xueqing Wu, Xuan Jing

**Affiliations:** Department of Reproductive Medicine Center, Children’s Hospital of Shanxi, The Affiliated Children’s Hospital of Shanxi Medical University, Shanxi Maternal and Child Health Hospital, Taiyuan 030001, China; Shanxi Center of Technology Innovation for Fertility Optimization, Reproductive Medicine Center, Children’s Hospital of Shanxi, Shanxi Maternal and Child Health Hospital, Taiyuan 030001, China; Yuncheng Children’s Hospital and Yuncheng Maternal, Child Health Hospital, Yuncheng 044000, China; Department of Nephrology, Shanxi Provincial People’s Hospital (Fifth Hospital), Shanxi Medical University, Taiyuan 030001, China; Department of Reproductive Medicine Center, Children’s Hospital of Shanxi, The Affiliated Children’s Hospital of Shanxi Medical University, Shanxi Maternal and Child Health Hospital, Taiyuan 030001, China; Department of Reproductive Medicine Center, Children’s Hospital of Shanxi, The Affiliated Children’s Hospital of Shanxi Medical University, Shanxi Maternal and Child Health Hospital, Taiyuan 030001, China; Department of Clinical Laboratory, Shanxi Provincial People’s Hospital, Shanxi Medical University, Taiyuan 030001, China; Department of Clinical Laboratory, Shanxi Provincial People’s Hospital, Shanxi Medical University, Taiyuan 030001, China; Shanxi University of Traditional Chinese Medicine, Taiyuan 030001, China; Department of Reproductive Medicine Center, Children’s Hospital of Shanxi, The Affiliated Children’s Hospital of Shanxi Medical University, Shanxi Maternal and Child Health Hospital, Taiyuan 030001, China; Department of Clinical Laboratory, Shanxi Provincial People’s Hospital, Shanxi Medical University, Taiyuan 030001, China; Department of Clinical Laboratory, Shanxi Provincial People’s Hospital, Shanxi Medical University, Taiyuan 030001, China; Department of Reproductive Medicine Center, Children’s Hospital of Shanxi, The Affiliated Children’s Hospital of Shanxi Medical University, Shanxi Maternal and Child Health Hospital, Taiyuan 030001, China; Department of Clinical Laboratory, Shanxi Provincial People’s Hospital, Shanxi Medical University, Taiyuan 030001, China; The School of Optical and Electronic Information, National Engineering Laboratory for Next Generation Internet Access System, Huazhong University of Science and Technology, Wuhan 430074, China; Department of Reproductive Medicine Center, Children’s Hospital of Shanxi, The Affiliated Children’s Hospital of Shanxi Medical University, Shanxi Maternal and Child Health Hospital, Taiyuan 030001, China; Shanxi Center of Technology Innovation for Fertility Optimization, Reproductive Medicine Center, Children’s Hospital of Shanxi, Shanxi Maternal and Child Health Hospital, Taiyuan 030001, China; Department of Clinical Laboratory, Shanxi Provincial People’s Hospital, Shanxi Medical University, Taiyuan 030001, China

**Keywords:** SIRT1, premature ovarian insufficiency, PKM2, lactate, granulosa cells

## Abstract

Premature ovarian insufficiency (POI) causes early loss of ovarian function, though its underlying metabolic-epigenetic mechanisms remain unclear. Using ovarian tissues from POI patients and a cisplatin-induced mouse model, we investigated the role of the NAD^+^-dependent deacetylase Sirtuin 1 (SIRT1) in granulosa cells. We found that SIRT1 expression was markedly downregulated in POI, correlating with hyperacetylation of the glycolytic enzyme pyruvate kinase M2 (PKM2) and pathogenic lactate accumulation. Restoring SIRT1 activity via intra-ovarian AAV-SIRT1 delivery or systemic administration of nicotinamide mononucleotide (NMN) reversed these phenotypes. This intervention improved follicular development, reduced granulosa cell apoptosis, and normalized serum anti-Müllerian hormone and follicle-stimulating hormone levels. Mechanistically, SIRT1 directly interacts with and deacetylates PKM2, suppressing its activity and limiting lactate production. Our work defines a critical SIRT1–PKM2–lactate metabolic axis that maintains ovarian homeostasis. Targeting this axis via NAD^+^-boosting interventions, such as NMN, presents a promising novel therapeutic strategy for POI.

## Introduction

Premature ovarian insufficiency (POI) is a multifactorial reproductive disorder characterized by the loss of ovarian function before the age of 40 years [1–3]. It affects about 1% of women worldwide and is associated with infertility, hypoestrogenism, and elevated gonadotropins [4, 5]. While a range of etiological factors—including genetic, autoimmune, and iatrogenic causes—have been implicated, the molecular mechanisms underlying the decline of ovarian reserve remain incompletely understood [6–10]. Recent advances highlight the importance of metabolic dysregulation and epigenetic imbalance in the pathogenesis of ovarian aging and follicular dysfunction [11–13].

Sirtuin 1 (SIRT1), a nicotinamide adenine dinucleotide (NAD^+^)-dependent deacetylase, functions as a metabolic sensor that integrates nutrient signals with chromatin remodeling and transcriptional regulation [14–16]. It has been shown to play a critical role in delaying aging-related cellular processes across various tissues [17–19]. In the context of reproductive biology, SIRT1 has been implicated in folliculogenesis, oocyte quality control, and granulosa cell (GC) survival [20–22]. Notably, SIRT1 expression decreases with age and is downregulated in several pathological models of ovarian insufficiency, such as those induced by chemotherapeutic agents or environmental toxins [23, 24].

Pyruvate kinase M2 (PKM2), a key enzyme in aerobic glycolysis, catalyzes the final step in the conversion of phosphoenolpyruvate to pyruvate [25, 26]. Acetylation of PKM2 enhances its enzymatic activity and promotes the accumulation of lactate—a metabolic byproduct that also acts as a signaling molecule to drive inflammation, senescence, and apoptosis [27, 28]. Although the deacetylation of PKM2 by SIRT1 has been characterized in extra-gonadal tissues [29–31], the functional consequence of this interaction is highly context-dependent. The ovary, with its exceptionally high metabolic demands for folliculogenesis, represents a unique physiological setting. Thus, investigating whether and how the SIRT1–PKM2 axis governs metabolic homeostasis in this specialized organ is crucial for understanding the fundamental mechanisms of ovarian aging.

Given the high metabolic demand of ovarian follicles and the reliance of GCs on glycolysis, we posited that dysregulation of the SIRT1–PKM2 axis is a key contributor to POI pathogenesis. Here, we demonstrate through integrated analyses of patient samples, POI models, and single-cell transcriptomics that SIRT1 maintains ovarian homeostasis by deacetylating PKM2 to suppress lactate production, thereby ensuring GC survival and follicular integrity. Beyond elucidating this mechanism, we show that its therapeutic activation—via NAD^+^ precursor supplementation with nicotinamide mononucleotide (NMN)—effectively re-establishes metabolic balance and rescues ovarian function *in vivo*, thereby nominating this pathway as a promising target for POI intervention.

## Results

### Decreased SIRT1 expression correlates with GC apoptosis in POI

**Figure 1.**
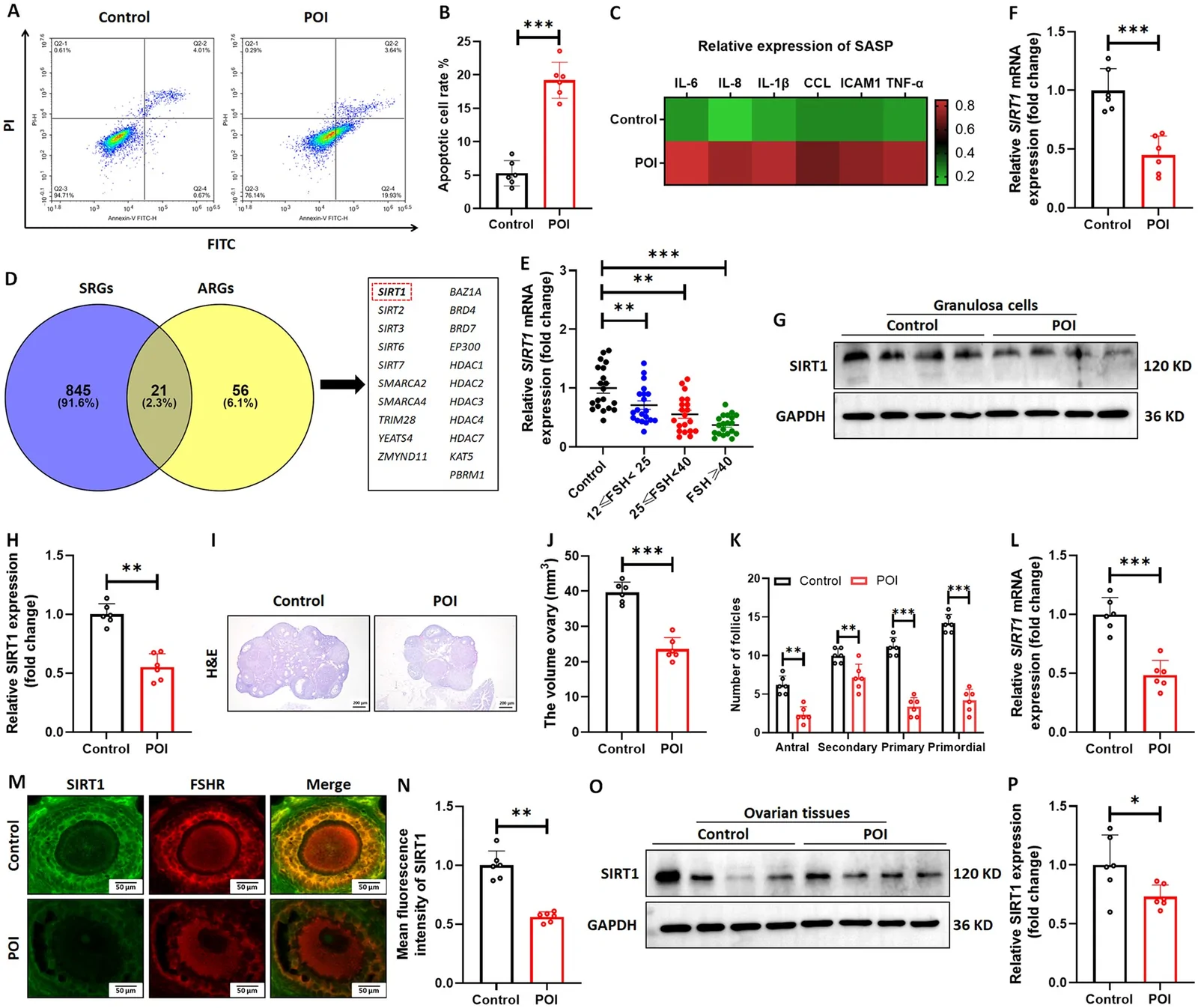
Decreased SIRT1 expression is associated with granulosa cell apoptosis and ovarian aging in POI. (A, B) Flow cytometry reveals increased apoptosis in GCs from POI patients compared to controls. (C) qRT-PCR shows elevated SASP marker expression in POI GCs. (D) Venn analysis identifies 21 acetylation regulators overlapping with 866 senescence-related genes. (E) SIRT1 expression in PBMCs negatively correlates with FSH levels, showing significant decreases in patients with elevated FSH. (F–H) *SIRT1* mRNA and protein levels are reduced in GCs from POI patients. (I–P) In a cisplatin-induced POI mouse model, SIRT1 expression is similarly downregulated in ovarian tissue, as shown by qRT-PCR, immunofluorescence (*n* = 6 mice per group), and Western blot (*n* = 4 mice per group). Data are shown as mean ± SD. *P < 0.05, **P < 0.01, ***P < 0.001.

GCs were isolated from the ovaries of women with POI and unaffected controls. Compared to controls, GCs from the POI group exhibited a significant increase in apoptosis (*P < *0.001) (Fig. 1A and 1B). Additionally, the expression of senescence-associated secretory phenotype (SASP) markers was notably higher in POI GCs (Fig. 1C). To explore the role of epigenetic regulation, particularly acetylation, in ovarian aging, we performed an intersection analysis of senescence-related genes (SRGs) and acetylation-related genes (ARGs) (Fig. 1D). From 866 SRGs obtained from the CellAge database, 21 key acetylation regulators were identified as potential mediators of cellular senescence. SIRT1, a key acetylation regulator, emerged as the focus of this study due to its high expression in ovarian tissues and its known role in regulating reproductive aging. SIRT1, as an NAD^+^ sensor, coordinates energy metabolism and epigenetic remodeling, making it a critical factor in ovarian aging. Furthermore, SIRT1 expression in peripheral blood mononuclear cells (PBMCs) significantly decreased across groups with rising follicle-stimulating hormone (FSH) levels (Fig. 1E). A significant inverse correlation was observed between serum FSH levels and *SIRT1* mRNA expression in PBMCs across all subjects (Fig. S1, P < 0.0001). In POI patients, *SIRT1* RNA and protein levels were significantly reduced in GCs (*P < *0.01) (Fig. 1F–H). Similarly, in the cisplatin-induced POI mouse model, SIRT1 expression was significantly lower in both ovarian tissue (*P < *0.01) (Fig. 1I–P). These findings suggest that reduced SIRT1 expression is closely linked to POI pathogenesis, potentially contributing to increased GC apoptosis and ovarian dysfunction.

### Ovary-specific SIRT1 upregulation alleviates ovarian dysfunction in a POI mouse model

**Figure 2.**
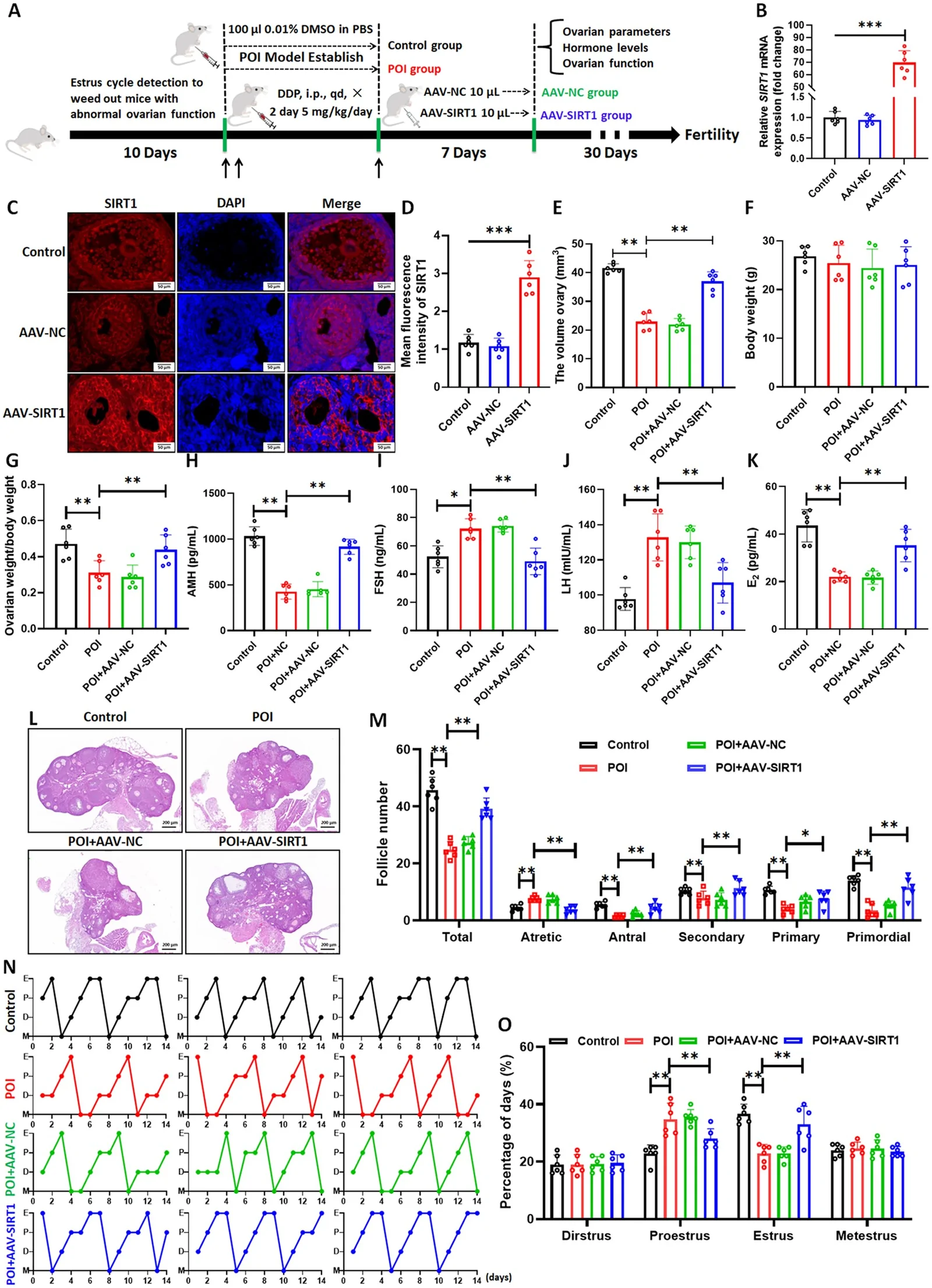
Ovary-specific SIRT1 overexpression improves ovarian function in POI mice. (A) Diagram showing intra-ovarian injection of AAV-SIRT1 or control AAV-NC. (B–D) qRT-PCR and immunofluorescence staining confirm increased SIRT1 expression in AAV-SIRT1-treated ovaries. (E–G) SIRT1 overexpression improves ovarian size and organ index. (H–K) SIRT1 overexpression significantly restored serum AMH and E2 levels and reduced FSH and LH levels. (L, M) Histology and follicle counts indicate increased healthy follicles and fewer atretic follicles. (N, O) Estrous cycle analysis. The Y‑axis represents the percentage of time spent in each estrous stage: P (proestrus), E (estrus), M (metestrus), and D (diestrus). The X‑axis represents time in days. AAV‑SIRT1 treatment significantly increased the proportion of proestrus and estrus phases compared with the POI+AAV‑NC group, indicating restoration of the estrous cycle. Data are shown as mean ± SD. *P < 0.05, **P < 0.01, ***P < 0.001.

Although SIRT1 downregulation was observed in both POI patients and cisplatin-induced POI mouse models, and its circulating levels declined with age, the causal relationship between ovarian SIRT1 loss and POI pathogenesis remained unclear. To clarify this, we performed intra-ovarian injections of AAV-SIRT1 or control AAV-NC (Fig. 2A), and confirmed that *SIRT1* mRNA and protein expression were significantly elevated in the AAV-SIRT1 group **(**Fig. 2B–D). Next, we evaluated whether SIRT1 overexpression could rescue ovarian phenotypes in the POI model. Compared with the POI group, AAV‑SIRT1 treatment significantly increased ovarian size and organ index. Similar effects were observed compared with the POI + AAV‑NC group. In contrast, no significant difference in body weight was detected among the three groups (Fig. 2E–G). Hormone profiling indicated that POI mice exhibited reduced anti-Müllerian hormone (AMH) and E2 levels and elevated FSH and luteinizing hormone (LH) levels, while AAV-SIRT1 treatment significantly restored these hormone levels toward normal ranges (Fig. 2H–K). Histological analysis revealed that, compared with both the POI group and the POI+AAV-NC group, the POI+AAV-SIRT1 group exhibited a significant increase in total follicle numbers (including primordial, primary, secondary, and antral follicles) and a reduction in atretic follicles (Fig. 2L and 2M). These findings suggest that ovarian-specific SIRT1 overexpression can reverse cisplatin-induced ovarian structural damage. Furthermore, estrous cycle monitoring demonstrated that AAV-SIRT1-treated mice showed a notable recovery of proestrus and estrus phases compared to untreated POI mice, indicating improved ovarian function (Fig. 2N and 2O). Together, these data highlight the therapeutic potential of SIRT1 in restoring ovarian reserve and hormonal balance in POI.

### SIRT1 overexpression restores endocrine function and reduces apoptosis in POI GCs

**Figure 3.**
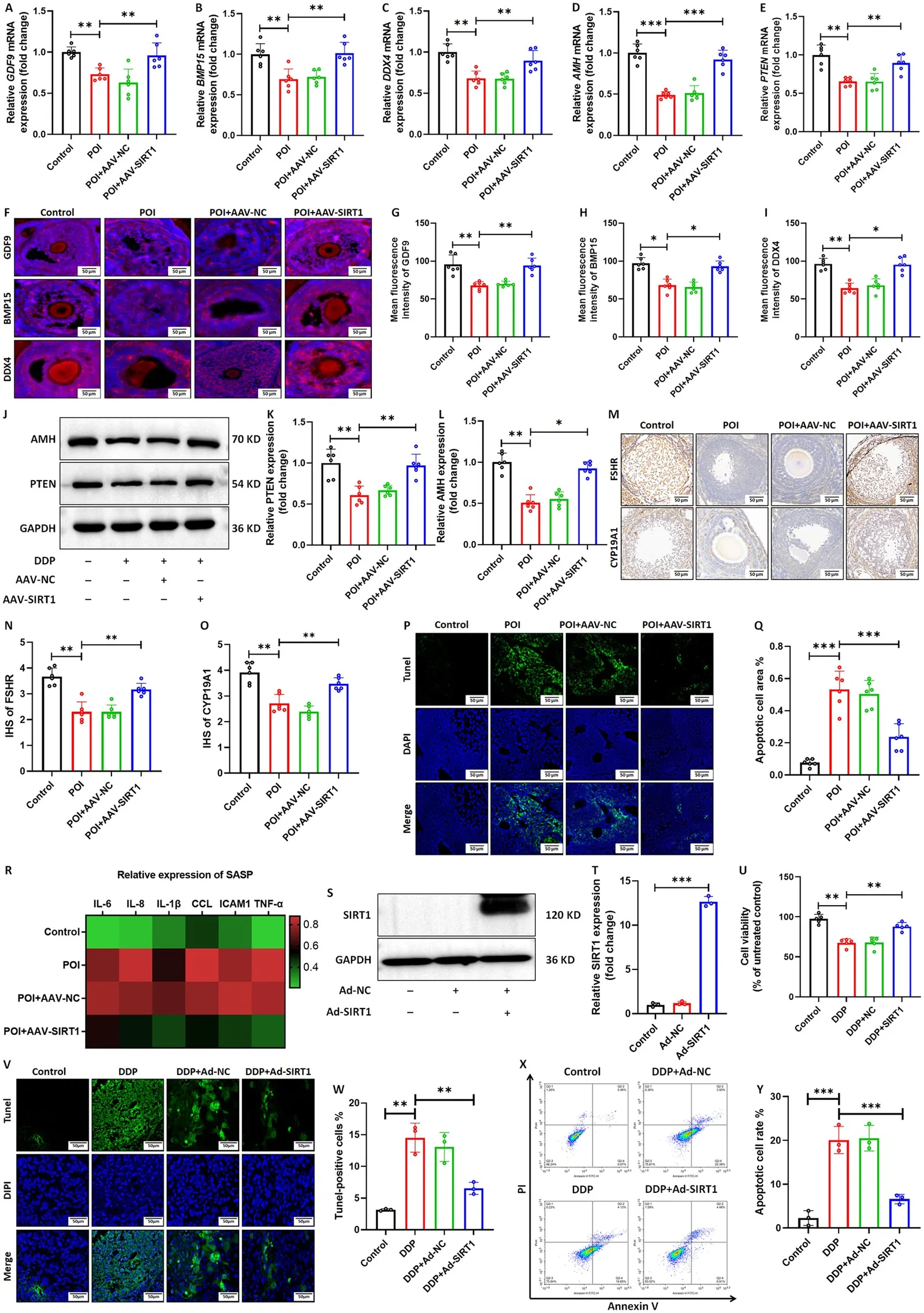
SIRT1 overexpression restores endocrine function and reduces apoptosis in POI granulosa cells. (A–C) qRT-PCR shows decreased expression of oocyte markers (*GDF9*, *BMP15*, *DDX4*) in POI ovaries, which is restored with AAV-SIRT1 treatment. (F–I) Representative immunofluorescence images of oocyte markers (GDF9, BMP15, DDX4) in ovarian sections. For DDX4, an oocyte-specific marker, intense staining is localized to oocytes. Some nonspecific background signal may be present in somatic compartments; this does not affect the qualitative assessment of oocyte presence, which shows a marked reduction in the POI group. (D, E, J–L) Granulosa cell markers (AMH, PTEN) are downregulated in POI ovaries, but restored following AAV-SIRT1 treatment. (M–O) AAV-SIRT1 treatment increases FSHR and CYP19A1 expression, indicating recovery of endocrine function. (P, Q) TUNEL assay shows significantly reduced apoptosis in AAV-SIRT1-treated granulosa cells compared to POI+AAV-NC group. (R) SASP marker expression is decreased in POI granulosa cells after AAV-SIRT1 treatment. (S, T) *In vitro*, adenovirus-mediated SIRT1 overexpression increases *SIRT1* mRNA and protein levels. (U) Cell viability assays show that SIRT1 overexpression improves GC viability and reduces DDP (cisplatin)-induced cytotoxicity. (V–Y) TUNEL and flow cytometry confirm reduced apoptosis in SIRT1-overexpressing GCs treated with cisplatin. Data are shown as mean ± SD. *P < 0.05, **P < 0.01, ***P < 0.001.

We assessed the expression of key oocyte-specific markers, including GDF9, BMP15, and DDX4, in ovarian tissue. In the POI mice, the mRNA (Fig. 3A–C) and protein (Fig. 3F–I) levels of these markers were significantly reduced, but they were restored following AAV-SIRT1 treatment. Similarly, GC-specific markers such as AMH and PTEN (Fig. 3D, 3E, and 3J–L) were decreased in POI ovaries, while their expression was significantly restored in the AAV-SIRT1-treated group. GCs play a crucial role in follicular development and estrogen synthesis. FSH receptor (FSHR), primarily expressed in GCs, mediates the response to FSH, promoting follicle growth and maturation. CYP19A1 is a key enzyme responsible for converting androgens into estrogens, maintaining estrogen levels, supporting follicular development, and ensuring proper reproductive function. Our study provides new evidence that AAV-SIRT1 treatment significantly upregulates the expression of FSHR and CYP19A1 in cisplatin-damaged ovaries (Fig. 3M–O), indicating that SIRT1 can restore the endocrine function of cisplatin-damaged ovarian tissue. To evaluate GC apoptosis, we performed a TUNEL assay. As shown in Fig. 3P and 3Q, apoptosis levels were significantly higher in the POI mice compared to the control group, confirming the harmful effects of cisplatin. However, AAV-SIRT1 treatment significantly reduced apoptosis, suggesting that SIRT1 has a protective effect on cisplatin-induced ovarian cell damage. Additionally, AAV-SIRT1 treatment significantly reduced the expression of SASP markers in POI GCs (Fig. 3R).

To validate these *in vitro* effects, we overexpressed SIRT1 in GCs using an adenovirus vector (Ad-SIRT1). Compared to the control group, cells treated with Ad-SIRT1 exhibited significantly increased *SIRT1* mRNA and protein levels, confirming successful overexpression (Fig. 3S and 3T). In this model, SIRT1 overexpression significantly enhanced GC viability, indicating that SIRT1 alleviates cisplatin-induced cell toxicity (Fig. 3U). Finally, TUNEL and flow cytometry analyses confirmed the protective effect of SIRT1. Cisplatin treatment significantly increased GC cell apoptosis (Fig. 3V–Y), whereas SIRT1 overexpression further significantly reduced apoptosis, highlighting its anti-apoptotic effect on ovarian GCs. In conclusion, our findings demonstrate that SIRT1 overexpression restores endocrine function in GCs, reduces cisplatin-induced apoptosis, and enhances GC viability.

### SIRT1 downregulation in aging GCs and its functional link to PKM2 inhibition

**Figure 4.**
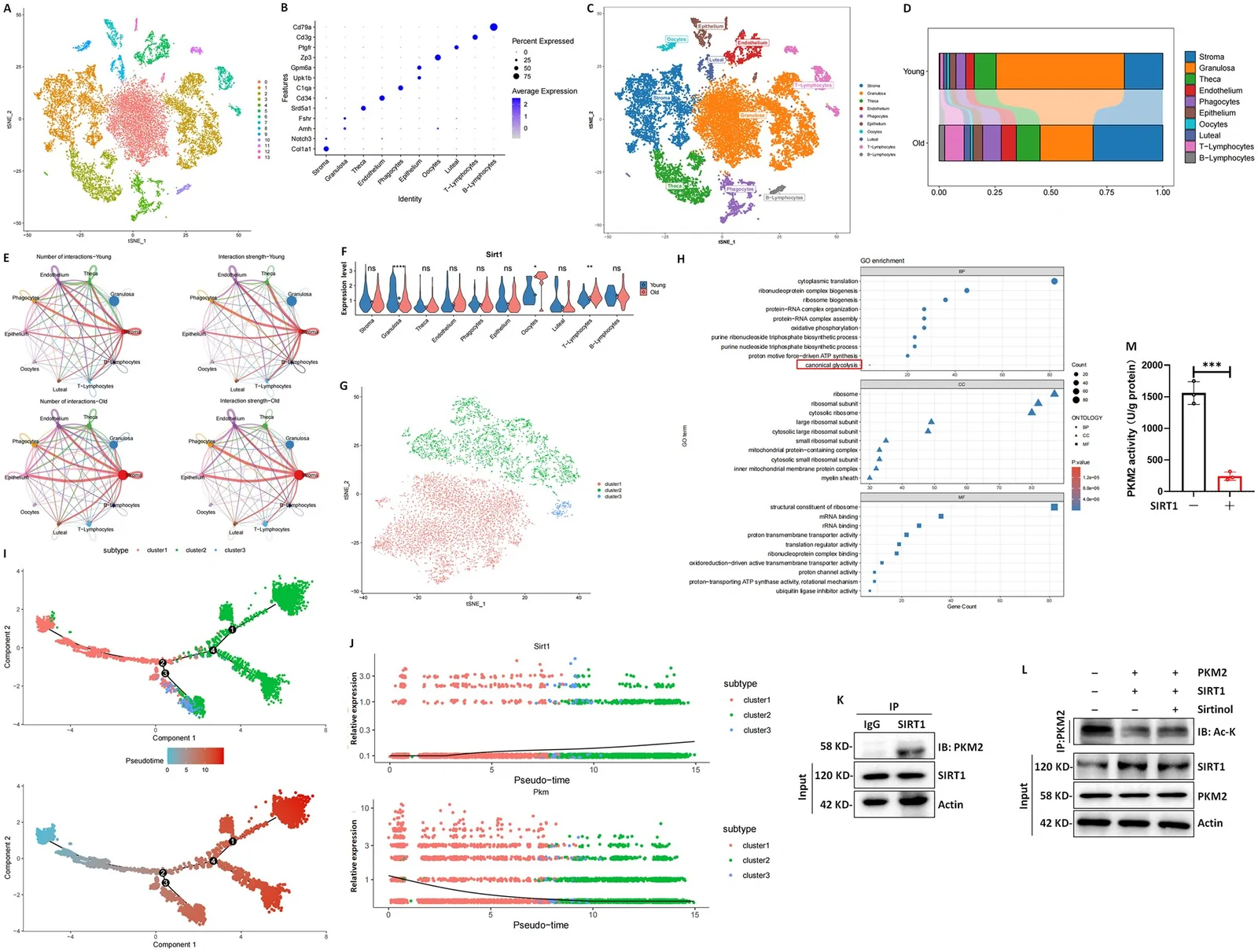
Aging-related downregulation of SIRT1 in granulosa cells is linked to PKM2 activity and glycolytic remodeling. (A) tSNE plot of scRNA-seq data (GSE232309) from young (3-month-old) and aged (9-month-old) mice, showing 14 distinct ovarian cell clusters. (B, C) Annotation of ovarian cell types based on marker genes, identifying major populations including granulosa cells. (D) Single-cell RNA sequencing revealed that in aged ovaries, the proportion of stromal cells increased whereas the proportion of granulosa cells decreased compared to young ovaries. (E) Ligand–receptor network analysis revealing altered intercellular communication between granulosa cells and other ovarian cell types in aged ovaries. (F) Violin plot showing significantly reduced SIRT1 expression in granulosa cells of aged mice compared to young controls. (G, H) Re-clustering of granulosa cells into three subclusters, with Cluster 1 enriched in glycolysis-related pathways based on GO analysis. (I, J) Pseudotime analysis shows reciprocal expression patterns of PKM2 (decreasing) and SIRT1 (increasing) along the granulosa cell differentiation trajectory. (K) Co-IP showing direct interaction between SIRT1 and PKM2 in granulosa cells transfected with a SIRT1-expressing plasmid. (L) Western blot demonstrating increased PKM2 acetylation upon SIRT1 inhibition by Sirtinol, indicating that SIRT1 deacetylates PKM2. (M) Functional assays confirm reduced PKM2 enzymatic activity with SIRT1 overexpression. *P < 0.05, **P < 0.01, ***P < 0.001.

To explore the underlying molecular mechanisms of SIRT1 function in ovarian aging, we reanalyzed scRNA-seq data (GSE232309) from ovarian tissues of young (3-month-old) and reproductively aged (9-month-old) mice. After principal components analysis dimensionality reduction, tSNE was used to cluster and visualize cells, resulting in 14 distinct clusters (Fig. 4A). Based on established marker genes [32], 10 major ovarian cell types were annotated, including stroma, granulosa, theca, endothelium, phagocytes, epithelium, oocytes, luteal cells, T lymphocytes, and B lymphocytes (Fig. 4B and 4C). Single-cell RNA sequencing revealed that in aged ovaries, the proportion of stromal cells increased whereas the proportion of GCs decreased compared to young ovaries (Fig. 4D). Ligand–receptor network inference revealed significant differences in intercellular communication between GCs and other cell types in aged versus young ovaries (Fig. 4E). Notably, SIRT1 expression was significantly decreased in GCs of aged mice, whereas it was significantly increased in oocytes; no significant changes were observed in other somatic cell types (Fig. 4F), prompting us to focus on this population for further analysis. This GC-specific decline of SIRT1 aligns with the immunofluorescence pattern observed in our POI model (Fig. 1M).

GCs were further reclustered into three subclusters (cluster 1–3) based on secondary tSNE analysis (Fig. 4G). GO enrichment analysis of subcluster-specific marker genes indicated that cluster 1 showed enrichment for terms including “glycolytic process” (Fig. 4H). While this bioinformatics result served as a preliminary indicator, it prompted us to investigate the key glycolytic regulator PKM2. Our subsequent functional experiments provided direct mechanistic evidence for its role (see below). Among glycolytic enzymes, PKM2 stood out due to its critical role in regulating glycolytic flux. Pseudotime analysis using 2,000 highly variable genes traced the differentiation trajectory of GCs from cluster 1 to clusters 2 and 3 (Fig. 4I). During this transition, PKM2 expression progressively declined, whereas SIRT1 expression increased (Fig. 4J), suggesting a reciprocal regulatory relationship.

To test whether SIRT1 directly interacts with PKM2, we conducted co-immunoprecipitation (co-IP) in GCs transfected with a SIRT1-expressing plasmid. PKM2 was successfully pulled down by anti-SIRT1 antibody, confirming their physical interaction (Fig. 4K). Furthermore, SIRT1 inhibition by sirtinol significantly increased PKM2 acetylation, indicating that SIRT1 promotes PKM2 deacetylation (Fig. 4L). Functional assays confirmed that SIRT1 reduces PKM2 enzymatic activity (Fig. 4M). Functional assays confirmed that SIRT1 reduces PKM2 enzymatic activity. Taken together, these data identify PKM2 as a downstream target of SIRT1 in GCs and suggest that SIRT1 suppresses PKM2 activity through deacetylation, thereby linking metabolic regulation to ovarian aging.

### Exogenous lactate induces ovarian dysfunction and follicular damage in a mouse model

**Figure 5.**
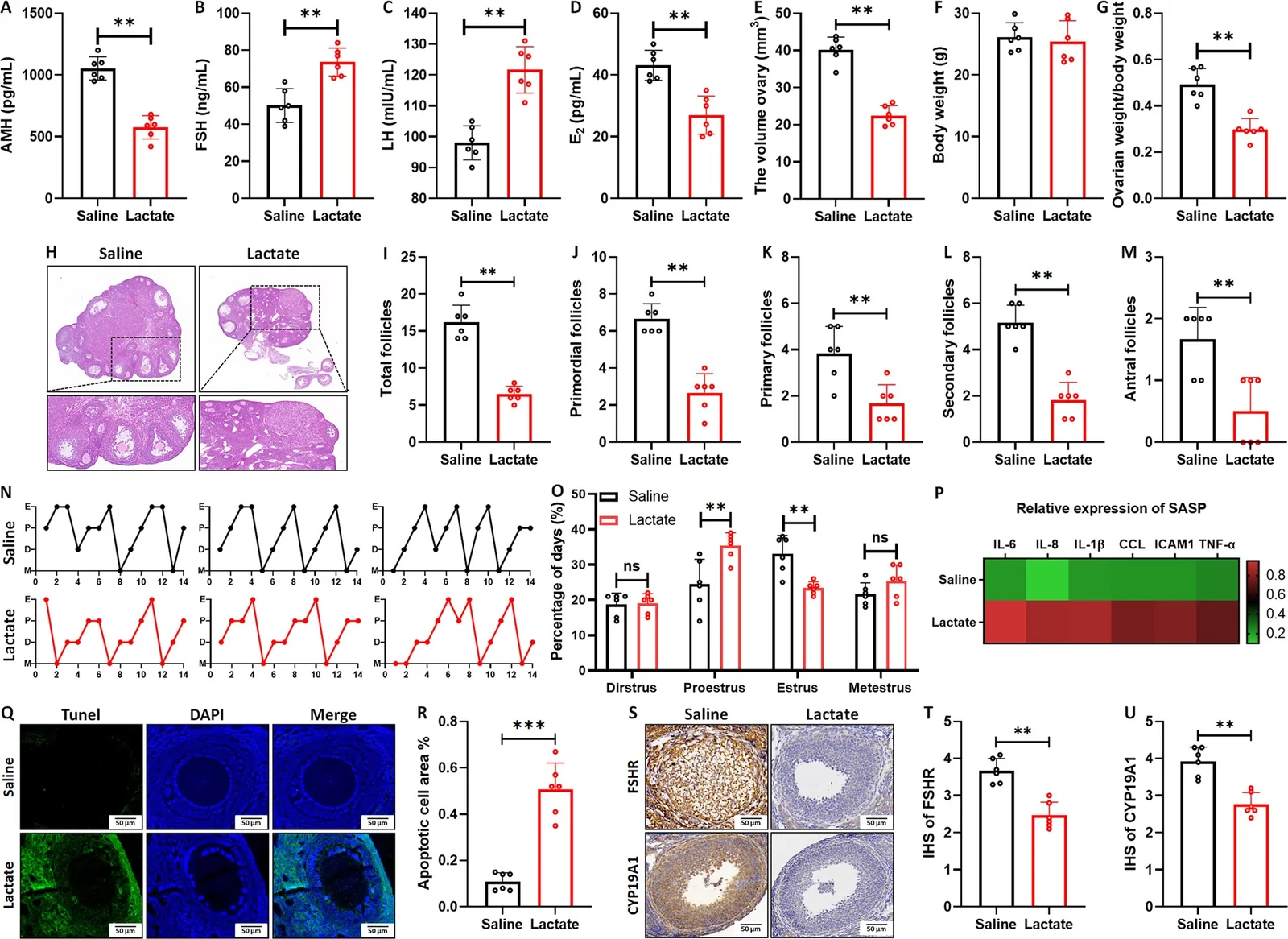
Exogenous lactate induces ovarian dysfunction and granulosa cell damage *in vivo*. (A–D) Serum hormone levels show that lactate-treated mice have decreased AMH and E2 and increased FSH and LH, indicating disrupted endocrine function. (E–G) Ovarian morphology and organ index measurements reveal reduced size and weight after lactate injection. (H–M) Histological analysis shows a significant decline in total follicles—including primordial, primary, secondary, and antral—in the lactate group. (N, O) Estrous cycle analysis indicates longer proestrus and shorter estrus phases following lactate treatment, suggesting impaired reproductive cycling. (P) qRT-PCR shows increased SASP marker expression in granulosa cells of lactate-injected mice. (Q, R) TUNEL assay confirms elevated granulosa cell apoptosis after lactate exposure. (S–U) Expression of FSHR and CYP19A1 is significantly reduced in ovaries from lactate-treated mice, indicating impaired granulosa cell endocrine function. Data are presented as mean ± SD. *P < 0.05, **P < 0.01, ***P < 0.001.

PKM2 is a key enzyme in glycolysis, and its increased expression and activity can lead to lactate accumulation in the ovaries, potentially contributing to the pathogenesis of POI. To investigate whether exogenous lactate can induce POI *in vivo*, we performed intraperitoneal lactate injections in mice and assessed the subsequent ovarian function. Hormonal profiling revealed that lactate infusion significantly reduced AMH and E2 levels while elevating FSH and LH levels (Fig. 5A–D). Compared with the saline-treated group, the lactate-treated mice exhibited significantly smaller ovarian size and lower organ index. In contrast, body weight did not differ significantly between the two groups (Fig. 5E–G). Histological analysis further demonstrated a significant decrease in the total number of follicles, including primordial, primary, secondary, and antral follicles in the lactate injection group (Fig. 5H–M). Estrous cycle monitoring revealed that lactate-treated mice showed an elevated proestrus phase and a reduced estrus phase compared to saline-injected mice, indicating compromised ovarian function (Fig. 5N and 5O). Additionally, lactate injection led to a significant increase in the expression of SASP markers in GCs (Fig. 5P), suggesting that lactate induces cellular senescence. As shown in Fig. 5Q and 5R, apoptosis levels were significantly higher in lactate-treated mice compared to the saline group, confirming the detrimental effects of lactate on ovarian GCs. Our study also found that lactate treatment significantly downregulated the expression of FSHR and CYP19A1 in ovarian tissues (Fig. 5S–U), indicating that lactate negatively impacts the endocrine function of the ovaries.

### Elevated PKM2 expression aggravates ovarian dysfunction in POI

**Figure 6.**
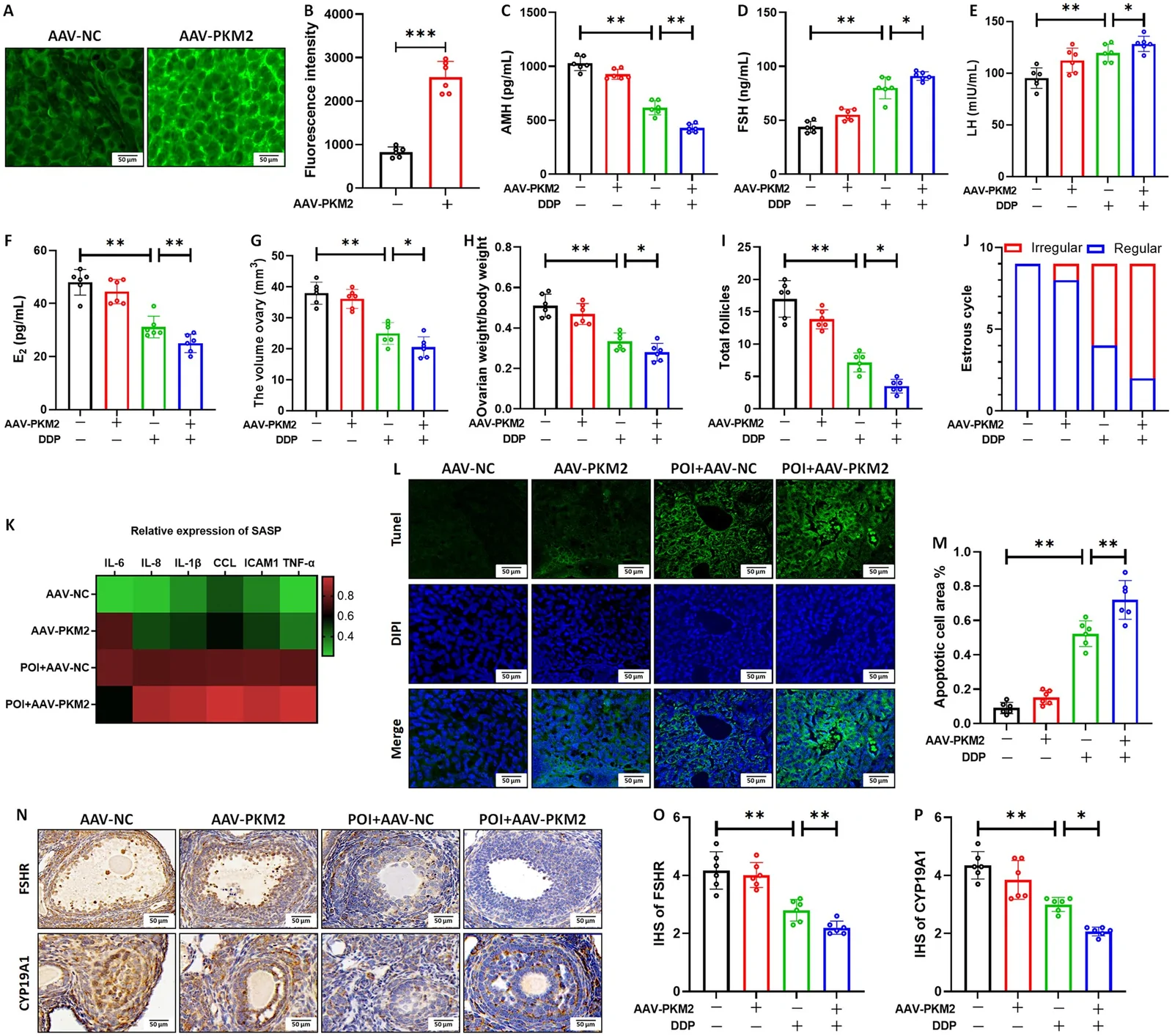
Elevated PKM2 expression worsens ovarian dysfunction in POI. (A, B) Immunofluorescence shows increased PKM2 expression in ovaries following AAV-PKM2 injection. (C–F) Hormonal analysis reveals reduced AMH and E2, and elevated FSH and LH levels in AAV-PKM2-treated mice, indicating exacerbated endocrine dysfunction. (G, H) AAV-PKM2-treated mice have smaller ovaries and lower organ index compared to controls. (I) Histological analysis shows further reduction in total follicle counts in AAV-PKM2 ovaries. (J) Estrous cycle monitoring reveals more irregularities in PKM2-overexpressing mice. (K) Increased SASP marker expression in granulosa cells is observed with PKM2 overexpression. (L, M) TUNEL and flow cytometry show significantly higher apoptosis in granulosa cells in the AAV-PKM2 group. (N–P) PKM2 overexpression further decreases FSHR and CYP19A1 expression in granulosa cells, indicating impaired ovarian function. Data are presented as mean ± SD. *P < 0.05, **P < 0.01, ***P < 0.001.

To further confirm the pathogenic role of excess lactate in POI progression, we increased PKM2 expression in the ovaries via *in situ* injection of AAV-PKM2, aiming to enhance lactate production. Immunofluorescence analysis confirmed a marked increase in PKM2 expression in ovarian tissues following AAV-PKM2 injection (Fig. 6A and 6B). The overexpression of PKM2 further exacerbated cisplatin-induced hormonal imbalances, as evidenced by a significant reduction in AMH and E2 levels, along with elevated FSH and LH levels (Fig. 6C–F). Mice in the AAV-PKM2 group also exhibited reduced ovarian size and lower organ index (Fig. 6G and 6H), and histological examination revealed a more pronounced decrease in total follicle counts (Fig. 6I). Additionally, PKM2 overexpression intensified disruptions in estrous cyclicity, with greater irregularities observed in the menstrual cycle patterns (Fig. 6J). Increased expression of SASP markers (Fig. 6K) and heightened GC apoptosis (Fig. 6L and 6M) were also observed, further supporting the deleterious effect of elevated PKM2 activity. Importantly, FSHR and CYP19A1 expression in GCs was further downregulated in AAV-PKM2-treated mice, indicating a greater impairment of ovarian endocrine function (Fig. 6N–P). Together, these findings demonstrate that abnormally elevated PKM2 promotes lactate accumulation and accelerates ovarian aging processes, reinforcing its role as a metabolic driver in POI pathogenesis.

### NMN restores NAD^+^/SIRT1 axis and ameliorates ovarian dysfunction in POI mice

**Figure 7.**
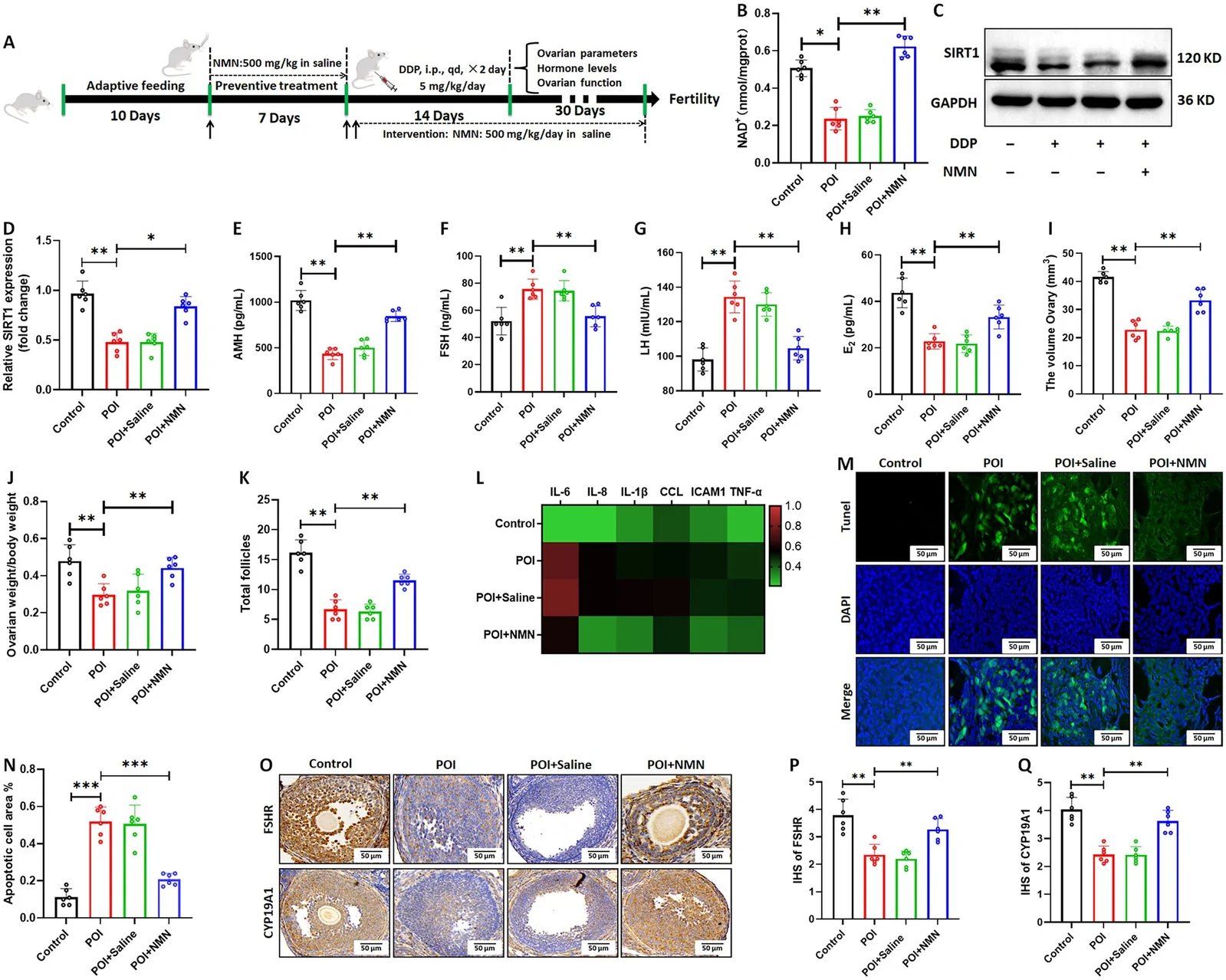
NMN restores NAD^+^/SIRT1 signaling and improves ovarian function in POI mice. (A) Schematic of NMN administration in the cisplatin-induced POI model. (B) NMN restores NAD^+^ levels significantly in POI ovaries. (C, D) *SIRT1* mRNA and protein levels are upregulated in NMN-treated ovaries. (E–H) NMN partially corrects hormone imbalances, increasing AMH and E2 while reducing FSH and LH. (I–K) NMN improves ovarian size, organ index, and total follicle count. (L) SASP marker expression is reduced in granulosa cells following NMN treatment. (M, N) TUNEL staining shows decreased granulosa cell apoptosis after NMN administration. (O–Q) Expression of FSHR and CYP19A1 is restored in granulosa cells, indicating improved endocrine function. Data are shown as mean ± SD. *P < 0.05, **P < 0.01, ***P < 0.001.

SIRT1 functions as a metabolic sensor and NAD^+^-dependent deacetylase. Given that PKM2 activation is promoted by acetylation and that SIRT1 exerts inhibitory effects via deacetylation, we explored whether restoring NAD^+^ levels could modulate SIRT1 activity and impact POI pathology. To this end, NMN, a key NAD^+^ precursor synthesized from NAMPT and PRPP, was administered to cisplatin-induced POI mice (Fig. 7A). We found that NAD^+^ levels were significantly decreased in the POI group compared to controls, but were restored to near-normal levels following NMN treatment (Fig. 7B). Consistently, SIRT1 expression was markedly reduced in POI ovaries and significantly upregulated after NMN administration (Fig. 7C and 7D). Hormonal profiling showed that NMN treatment partially restored the altered hormone levels in POI mice, with AMH, E2, FSH, and LH approaching physiological ranges (Fig. 7E–H). Moreover, NMN administration improved ovarian morphology, including increased ovarian volume, improved ovarian organ index, and a higher total follicle count (Fig. 7I–K). We also observed a reduction in SASP marker expression following NMN treatment, suggesting attenuation of senescence-associated signaling (Fig. 7L). Apoptosis in GCs was significantly reduced by NMN, as shown by TUNEL analysis (Fig. 7M and 7N). In parallel, NMN restored the expression of FSHR and CYP19A1 in the ovarian GCs, indicating a functional recovery of the ovarian endocrine axis (Fig. 7O–Q). Collectively, these results demonstrate that NMN supplementation rescues NAD^+^ levels, activates SIRT1, and counteracts the hormonal imbalance, follicular atrophy, and endocrine dysfunction observed in POI, supporting a metabolic mechanism involving SIRT1–PKM2 regulation.

## Discussion

**Figure 8.**
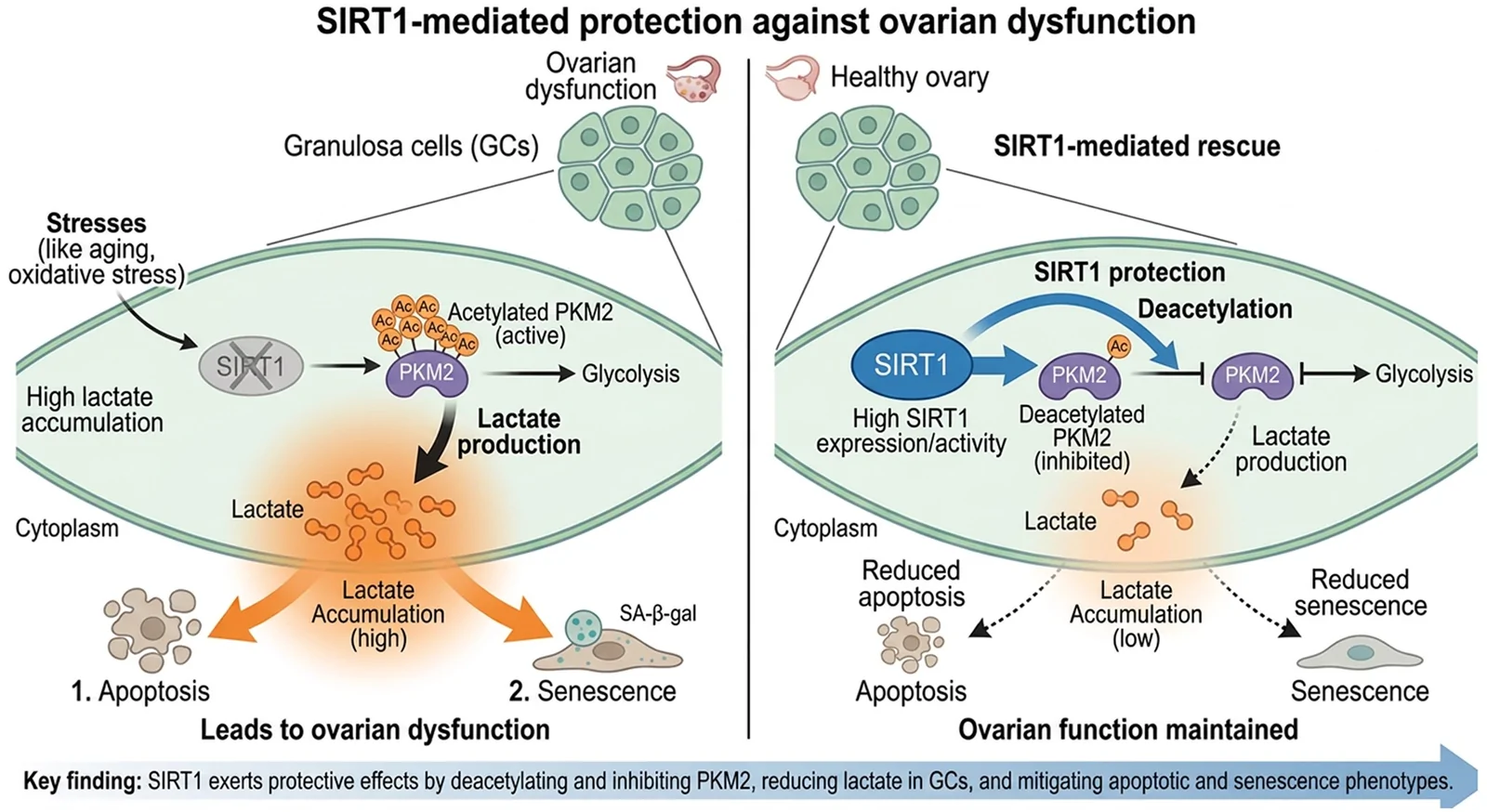
Schematic diagram of the SIRT1–PKM2–lactate signaling axis in ovarian granulosa cells. The model highlights that the NAD^+^/SIRT1 pathway negatively regulates PKM2 activity via deacetylation, which in turn suppresses lactate-induced apoptosis.

In this study, we reveal a previously uncharacterized metabolic–epigenetic regulatory axis involving SIRT1 and PKM2 that modulates lactate homeostasis and ovarian reserve in the context of POI. Through a combination of human sample analysis, murine POI models, single-cell transcriptomics, and targeted metabolic interventions, we demonstrate that SIRT1 exerts protective effects against ovarian dysfunction by deacetylating and inhibiting PKM2, thereby reducing lactate accumulation in GCs and mitigating associated apoptotic and senescence phenotypes (Fig. 8).

Our findings are consistent with recent reports implicating SIRT1 as a key regulator of PKM2 activity in nonreproductive systems. For instance, Lian et al. [29] showed that SIRT1-mediated deacetylation of PKM2 limits pathological lactate accumulation and neurotoxicity in Parkinsonism models, while Zhao et al. [30] demonstrated that pharmacologic activation of SIRT1 facilitates PKM2 degradation via autophagy in inflammatory injury models. Moreover, Chen et al. [31] identified nuclear PKM2 as a detrimental factor in LPS-induced lung injury, which was suppressed by SIRT1-dependent mechanisms. These data collectively underscore a conserved role for SIRT1 in restraining PKM2-driven metabolic stress, a paradigm we extend here to the ovary.

Mechanistically, we show that SIRT1 interacts directly with PKM2 in GCs and deacetylates it, leading to reduced enzymatic activity and lactate output. While the acetylation status of PKM2 in ovarian tissues of POI mice or following NMN treatment was not directly measured, the functional consequences of this regulatory axis are robustly supported. Our observation of pathological lactate accumulation in POI, coupled with the findings that both PKM2 overexpression and exogenous lactate administration are sufficient to recapitulate POI-like phenotypes, positions lactate overproduction as a key downstream effector. Exogenous lactate administration or AAV-mediated overexpression of PKM2 recapitulates POI-like phenotypes, including follicular depletion, endocrine disruption, and GC apoptosis, thereby functionally linking PKM2–lactate signaling to ovarian atrophy. Critically, restoring the upstream NAD^+^/SIRT1 node via NMN supplementation effectively rescued these downstream phenotypes. These results further support the notion that aberrant glycolytic reprogramming and lactate dysregulation may serve as upstream drivers of ovarian aging.

Importantly, restoration of SIRT1 activity through NAD^+^ supplementation with NMN significantly ameliorated POI phenotypes. This aligns with recent studies suggesting the therapeutic relevance of NAD^+^-boosting strategies in reproductive aging [33–35]. Given the high metabolic demand of GCs and their reliance on finely tuned glycolytic flux, the SIRT1–NAD^+^ axis may represent a central node in safeguarding follicular viability under both physiological and pathological conditions.

Our scRNA-seq reanalysis further provides transcriptomic evidence that SIRT1 expression declines in aged GCs, while PKM2 is enriched in glycolysis-related clusters, reinforcing their functional antagonism during GC maturation and senescence. This temporal expression pattern suggests a developmental switch in metabolic programming that may be dysregulated in POI.

### Research limitations

While this study offers mechanistic insights, several limitations remain. First, whether SIRT1–PKM2 interactions extend to other ovarian cell types, such as theca or stromal cells, warrants further investigation. Second, the involvement of nuclear versus cytoplasmic PKM2 pools and their downstream transcriptional targets in ovarian aging remains to be clarified. Third, the translational potential of NAD^+^ precursors or SIRT1 activators in clinical POI settings calls for rigorous longitudinal studies and optimization of delivery strategies. Fourth, while we focused on the metabolic consequence of PKM2 activation (lactate production), future studies should investigate whether PKM2 also exerts pro-aging effects in GCs through nuclear translocation and transcriptional co-activator functions, which would further expand the molecular network underlying ovarian aging.

In conclusion, our data highlight the SIRT1–PKM2–lactate pathway as a key determinant of ovarian metabolic homeostasis and reserve capacity. Targeting this axis via SIRT1 activation or lactate reduction may offer novel therapeutic strategies for preserving fertility and delaying ovarian aging in women at risk for or experiencing POI.

## Methods

### Research ethics

This study was conducted under the project titled “The role and mechanism of HuMSCs-Exos regulating histone demethylase KDM3A on mitochondrial integrity of ovarian GCs in premature ovarian failure.” All animal experiments were approved by the Institutional Animal Care and Use Committee of Shanxi Provincial People’s Hospital (Ethics Ref. No. 679) and were performed in accordance with the ethical guidelines of the International Council for Laboratory Animal Science (ICLAS). Human subject investigations complied with the Declaration of Helsinki and were approved by the Medical Ethics Committee of the Center for Reproductive Medicine at Shanxi Children’s Hospital (Ethics Ref. No. IRB-GKY-2020-008). Written informed consent was obtained from all participating patients prior to sample collection.

### Patient populations and peripheral blood collection

Patients diagnosed with POI were recruited from the Reproductive Medicine Center of Shanxi Children’s Hospital (Shanxi Maternal and Child Health Hospital). The diagnosis of POI was based on the 2016 guidelines of the European Society of Human Reproduction and Embryology (ESHRE), which define POI as the occurrence of primary or secondary amenorrhea lasting more than four consecutive months before the age of 40 years, along with elevated serum FSH levels exceeding 25 IU/L on at least two occasions more than four weeks apart. Age-matched healthy women undergoing routine physical examinations were enrolled as controls. A total of 80 participants were included and categorized into four groups according to serum FSH levels: control group (FSH ≤ 10 IU/L, *n *= 20), POI group 1 (FSH ≥ 40 IU/L, *n *= 20), POI group 2 (FSH = 20–40 IU/L, *n *= 20), and POI group 3 (FSH = 12–25 IU/L, *n *= 20). Peripheral blood samples were collected from all participants, and plasma FSH concentrations were measured using a chemiluminescent immunoassay [36]. This study was approved by the Institutional Review Board of the Reproductive Medicine Center of Shanxi Children’s Hospital (Ethics Ref. No. IRB-GKY-2020-008), and written informed consent was obtained from all participants after full explanation of the study purpose and procedures.

### Animals

All animal experiments were conducted in strict accordance with the “Guidelines for the Care and Use of Laboratory Animals” issued by the National Institutes of Health, China. The experimental protocol was approved by the Animal Care and Use Committee (IACUC) of Shanxi Provincial People’s Hospital. Female C57BL/6 mice, aged 6–8 weeks, were purchased from Jiangsu Jicui Yakang Biotechnology Co., Ltd. (license number: SCXK(Su)2023-0009). The mice were housed in a controlled environment with a temperature of 20°C–26°C, humidity of 40%–70%, and provided with *ad libitum* access to food and water.

### Cell culture

Human ovarian GCs were isolated from POI patients and control individuals. The cells were cultured in Dulbecco’s modified Eagle medium (DMEM) supplemented with 10% fetal bovine serum (FBS) and 1% penicillin-streptomycin. The cultures were maintained at 37°C in a humidified atmosphere with 5% CO_2_ [36]. Mouse GCs were isolated from the ovaries of 8-week-old female C57BL/6 mice purchased from Jiangsu Jicui Yakang Biotechnology Co., Ltd. (license number: SCXK(Su)2023-0009). Following cervical dislocation, ovaries were dissected and washed with sterile PBS. The tissues were digested with 0.1% hyaluronidase (Sigma, USA) at 37°C for 5 min. The resulting cell suspension was filtered through a 200-mesh sieve, washed with PBS, and resuspended in DMEM/F12 medium (Gibco, USA) containing 10% FBS and 1% penicillin-streptomycin. The cells were seeded in 6-well plates and incubated at 37°C with 5% CO_2_. After 24 h, the culture medium was replaced to remove nonadherent cells. The adherent GCs were cultured for subsequent experiments [37].

### Establishment and treatment of POI model mice

To induce POI, female C57BL/6 mice were intraperitoneally injected with cisplatin (5 mg/kg) on days 1 and 2, which selectively damages primordial and primary ovarian follicles (Fig. 2A). Control mice received an equal volume of vehicle (DMSO diluted with saline). Two weeks after the final cisplatin injection, the estrous cycle was assessed via vaginal cytology, and ovarian morphology was evaluated to confirm successful POI model establishment. After 7 days, mice in the AAV treatment groups received 10 µL of the respective AAVs (AAV-NC, AAV-SIRT1, or AAV-PKM2) via intra-ovarian injection. To assess the therapeutic effect of NMN, the POI+NMN group was administered NMN (500 mg/kg/day) by gavage starting on day 8 for 44 days, while the POI+Saline group received saline (Fig. 7A) [38, 39]. Mice were monitored daily for health and body weight, and at the end of the treatment, ovaries were collected for histological, immunofluorescence, and Western-blot analysis.

### Histology examination

Mice were euthanized, and cardiac blood was collected. Ovaries were excised, weighed, and fixed in 4% paraformaldehyde for 24 h. The fixed tissues were embedded in paraffin and sectioned at a thickness of 5 μm. For morphological analysis, every fifth section was stained with hematoxylin and eosin (H&E; Solarbio, Beijing, China). To quantify ovarian follicles, every fourth section from each set of five consecutive slices was selected for analysis. Follicles at different developmental stages—including primordial, primary, secondary, and atretic follicles—were counted according to the method of Pedersen and Peters (1968) using a double-blind approach [40, 41]. The average number of follicles was calculated for each ovary.

### Serum hormone measurement

Serum samples were collected from the heart ventricles of mice immediately after euthanasia. The samples were centrifuged at 3,000 rpm for 15 min at 4°C to separate the serum. The obtained serum was aliquoted and stored at −80°C until further analysis. Concentrations of AMH, estradiol (E2), FSH, and LH were measured in serum samples collected from mice in the diestrus phase using commercial enzyme-linked immunosorbent assay kits (Beijing North Institute of Biotechnology Co., Ltd.).

### Database collection and processing

In this study, 866 SRGs were obtained from the CellAge online database. These genes were intersected with key ARGs, resulting in the identification of 21 key acetylation regulators associated with cellular senescence. Single-cell RNA sequencing (scRNA-seq) data were downloaded from the GEO database (GSE232309), which included ovarian samples from eight wild-type mice: four young (3-month-old) and four reproductively aged (9-month-old). The sequencing platform was GPL24247 Illumina NovaSeq 6000. The dataset was reanalyzed and underwent quality control, dimensionality reduction, clustering, and cell-type annotation. Differential expression of PKM2 and SIRT1 across various ovarian cell subpopulations was examined. In addition, downstream analyses included cell–cell communication analysis, secondary clustering of GCs, Gene Ontology (GO) enrichment analysis, and pseudotime trajectory analysis.

### Detection of apoptosis

Apoptosis of GCs was assessed by flow cytometry using an Annexin V-FITC/PI apoptosis detection kit (BD Biosciences, San Jose, CA, USA) according to the manufacturer’s instructions. In addition, apoptosis in paraffin-embedded ovarian tissue sections and cell slides was detected using a TUNEL assay kit (Promega, Madison, WI, USA), following the manufacturer’s protocol.

### Quantitative real-time PCR

Total RNA was extracted from mouse ovarian tissues and cultured cells using TRIzol reagent (Invitrogen, USA). cDNA was synthesized using a reverse transcription kit (Roche, Switzerland). qRT-PCR was performed with a SYBR Green PCR kit (Roche, Switzerland) on a real-time PCR system. The cycling conditions were 95°C for 10 min, followed by 40 cycles of 95°C for 15 s, 60°C for 1 min, and 72°C for 30 s, with a final extension at 72°C for 5 min. Gene expression was normalized to GAPDH and calculated using the 2^−ΔΔCT^ method. Primer sequences are listed in Table 1.

**Table 1.** Sequences of primers used for fluorescence quantitative PCR in this study.

Gene	Primer sequence (forward)	Primer sequence (reverse)
***Human-SIRT1***	TAGCCTTGTCAGATAAGGAAGGA	ACAGCTTCACAGTCAACTTTGT
***Human-GAPDH***	AATGGATTTGGACGCATTGGT	TTTGCACTGGTACGTGTTGAT
*Mus-SIRT1*	TGATTGGCACCGATCCTCG	CCACAGCGTCATATCATCCAG
*GDF9*	GGCACAAAGGTTCAGGGGG	CACCCGGTCCAGGTTAAACA
*AMH*	CCACACCTCTCTCCACTGGTA	GGCACAAAGGTTCAGGGGG
*BMP15*	TGGGGAGTGGTGCTTTTTATG	GGGCAATGTAGGGTCGTCAG
*DDX4*	GAGAACACATCTACAACTGGTGG	CCTCGCTTGGAAAACCCTCT
*PTEN*	TGGATTCGACTTAGACTTGACCT	GCGGTGTCATAATGTCTCTCAG
*IL-18*	GTCACAGCCAGTCCTCTTACTTCAC	CCCTTGTCGAGAATGGGCAG
*IL-1β*	GACCAGAATGTGCCACGGTT	CTCGCCTGCTCATCAACAAG
*IL-6*	GTTCTCTGGGAAATCGTGGA	GGAAATTGGGGTAGGAAGGA
*TNF-α*	TATGGCCCAGACCCTCACA	GGAGTAGACAAGGTACAACCCATC
*CCL-2*	TTCTTCGATTTGGGTCTCCTTG	GTGCAGCTCTTGTCGGTGAA
*ICAM1*	GTGATGCTCAGGTATCCATCCA	CACAGTTCTCAAAGCACAGCG
*Mus-GAPDH*	AGGTCGGTGTGAACGGATTTG	TGTAGACCATGTAGTTGAGGTCA

### Immunofluorescence staining

Immunofluorescence staining was used to assess the expression of SIRT1, GDF9, BMP15, DDX4, and FSHR in frozen ovarian sections and GCs. Samples were fixed and incubated overnight at 4°C with primary antibodies: anti-SIRT1 (1:100, Abcam), anti-GDF9, anti-BMP15, anti-DDX4, and anti-FSHR (all 1:200, Proteintech or Affinity). The next day, samples were equilibrated to room temperature and incubated for 1 h with Alexa Fluor 488 or 594-conjugated goat anti-Rabbit IgG secondary antibodies (Abbkine). Nuclei were counterstained with DAPI for 10 min in the dark. Images were captured using a Leica inverted fluorescence microscope, and mean fluorescence intensity was analyzed with ImageJ.

### Western-blot analysis

GCs or ovarian tissues were lysed using RIPA buffer, and protein concentrations were measured with a BCA protein assay kit (Solarbio, China). Equal amounts of protein were separated by SDS–PAGE, transferred to PVDF membranes, and blocked with 5%–7% skim milk for 1 h at room temperature. Membranes were incubated overnight at 4°C with primary antibodies: anti-SIRT1 (1:2,000, Abcam), anti-AMH (1:2,000, Proteintech), anti-PTEN (1:2,000, Proteintech), anti-PKM2 (1:2,000, Cell Signaling Technology), anti-Acetylated Lysine (1:2,000, Cell Signaling Technology), anti-actin (1:20,000, Cell Signaling Technology), and anti-GAPDH (1:20,000, Proteintech). After washing three times with TBST, membranes were incubated with secondary antibodies (1:20,000) at 37°C for 1 h. Protein bands were visualized using an ECL reagent kit (Novland, China), and band intensities were quantified using ImageJ software.

### Co-immunoprecipitation

The co-IP assay was performed as previously described [29, 42]. GCs were transfected with plasmids expressing SIRT1 and PKM2 using Lipofectamine for 24 h, or treated with Sirtinol, while control cells were collected. Cells were lysed in co-IP lysis buffer containing 50 mmol/L Tris–HCl (pH 7.5), 150 mmol/L NaCl, 5% glycerol, 0.5% Triton X-100, and protease inhibitors (Thermo, Cat No. 88805). Protein extracts were prepared for IP and western blotting, and incubated with magnetic beads-conjugated antibodies overnight at 4°C on a rotator. The immune complexes were then analyzed by SDS–PAGE followed by western blotting using the appropriate primary antibodies.

### Preparation of recombinant adenoviruses

Recombinant adenoviruses were used to express SIRT1 and PKM2 as previously described [29]. Mice were injected with AAV-SIRT1, AAV-PKM2, or control AAV-NC. The viruses were constructed by HanBio (Shanghai, China). Each mouse received 4 mL of virus at a concentration of 1 × 10^12^ viral genomes (VG)/mL. One month after injection, mice were used for PD model generation. Full-length mouse *SIRT1* or *PKM2* genes were cloned into the pAd-TrackCMV shuttle vector and linearized with PmeI. The plasmids were transformed into BJ5183 cells with the pAdEasy-1 adenoviral backbone. Recombinant clones were selected by kanamycin resistance and confirmed by restriction enzyme analysis. After digestion with PacI, the plasmids were transfected into HEK-293A cells. Recombinant adenoviruses were generated within 14–20 days, purified by cesium chloride density gradient centrifugation, and dialyzed in PBS with 10% glycerol for stability.

### NAD^+^ assay

NAD^+^ levels were determined using the Coenzyme I NAD(H) content test kit (Nanjing Jiancheng, China) according to the manufacturer’s protocol. In each experiment, at least 5 × 10^6^ cells were used for total NAD^+^ extraction. Absorbance at 570 nm was measured using a standard enzyme instrument (Bio-Tek Instruments, Inc., Winooski, VT, USA). NAD^+^ concentrations in the samples were calculated following the provided formula.

### Immunohistochemistry analysis

Ovaries were fixed in 4% formaldehyde, embedded in paraffin, and sectioned at 5 μm. Sections were treated with 3% hydrogen peroxide for 30 min, then boiled in 0.1 mol/L sodium citrate (pH 6.0) for antigen retrieval. After permeabilization with 1% Triton X-100 in PBST for 30 min, sections were blocked with 5% bovine serum albumin for 45 min. Sections were incubated overnight at 4°C with primary antibodies: anti-FSHR (1:200, Proteintech) and anti-CYP19A1 (1:200, Proteintech). After washing, sections were incubated with secondary antibodies for 90 min at room temperature, followed by HRP for 45 min. Signal was visualized using DAB, with tan staining indicating a positive result. Negative controls used PBST instead of primary antibodies. Stained sections were observed under a light microscope.

### Pyruvate kinase activity assay

GCs were transfected with a SIRT1-expressing plasmid, and after 24 h, cells were harvested for PKM2 protein purification. PKM2 was extracted using a protein extraction buffer, and the concentration was measured using a BCA protein assay kit. Pyruvate kinase activity was measured using the Pyruvate Kinase Activity Assay Kit (Sigma-Aldrich, Cat. No. MAK072). The reaction system consisted of 50 μL purified PKM2 mixed with 50 μL reaction mix containing PEP, ADP, NADH, and LDH. Absorbance at 340 nm was recorded for 20 min at 37°C using a microplate reader. PKM2 activity was calculated based on the change in absorbance and normalized to the protein concentration (units of activity per g of protein).

### Quantification and statistical analysis

Data are expressed as the mean ± SD. Statistical significance between two groups was assessed using a two-tailed Student’s *t*-test. For comparisons among multiple groups, statistical analysis was performed using either a *t*-test or one-way analysis of variance (ANOVA). All statistical analyses were conducted using GraphPad Prism version 9.0.0. Statistical significance was accepted at *P *< 0.05.

## Supplementary Material

lnag022_Supplementary_Data

## Data Availability

All the raw datasets generated during the current study are available from the corresponding author on reasonable request.
