# Supplementary material for: SIRT1 deacetylates PKM2 to constrain lactate production and protect against premature ovarian insufficiency
Source: Life Med. 2026 Jun 9;5(4):lnag022. doi: 10.1093/lifemedi/lnag022 (PMC13387601; doi:10.1093/lifemedi/lnag022)
Supplement: lnag022_Supplementary_Data [file lnag022_supplementary_data.zip › SI_upload.docx]

**SIRT1 deacetylates PKM2 to constrain lactate production and protect against premature ovarian insufficiency**

**Xiangrong Cui¹^,^³^,^⁷^,#^, Chongyang Han⁶^,#^, Puhua Zhang¹, Ruotong Ju¹, Ruixiang Zhu², Jiali Luo², Li Peng⁴, Chenyu Jia¹, Xinyu Zhu², Shu Wang², Huihui Li¹, Tingting Xue², Meng Zou⁵^,*^**, Xueqing Wu¹^,^³^,*^**, Xuan Jing²^,^**^*^

¹Department of Reproductive Medicine Center, Children’s Hospital of Shanxi, The Affiliated Children’s Hospital of Shanxi Medical University, Shanxi Maternal and Child Health Hospital, Taiyuan 030001, China
²Department of Clinical Laboratory, Shanxi Provincial People’s Hospital, Shanxi Medical University, Taiyuan 030001, China
³Shanxi Center of Technology Innovation for Fertility Optimization, Taiyuan 030001, China
⁴Shanxi University of Traditional Chinese Medicine, Taiyuan 030001, China
⁵The School of Optical and Electronic Information, National Engineering Laboratory for Next Generation Internet Access System, Huazhong University of Science and Technology, Wuhan 430074, China
⁶Department of Nephrology, Shanxi Provincial People's Hospital (Fifth Hospital) of Shanxi Medical University, Taiyuan 030001, China
⁷Yuncheng Children's Hospital and Yuncheng Maternal and Child Health Hospital, Yuncheng 044000, China

^#^These authors contributed equally to this work.

^*^Correspondence: jx05070103@163.com (X.J.), xueqingwu416@163.com (X.W.), zoumeng@hust.edu.cn (M.Z.)

**Supplement Figure S1. A significant inverse correlation was observed between serum FSH levels and SIRT1 mRNA expression in PBMCs across all subjects (Fig. S1, *p* < 0.0001 ).**
