# Supplementary figures and images for: SIRT1 deacetylates PKM2 to constrain lactate production and protect against premature ovarian insufficiency
Source: Life Med. 2026 Jun 9;5(4):lnag022. doi: 10.1093/lifemedi/lnag022 (PMC13387601; doi:10.1093/lifemedi/lnag022)

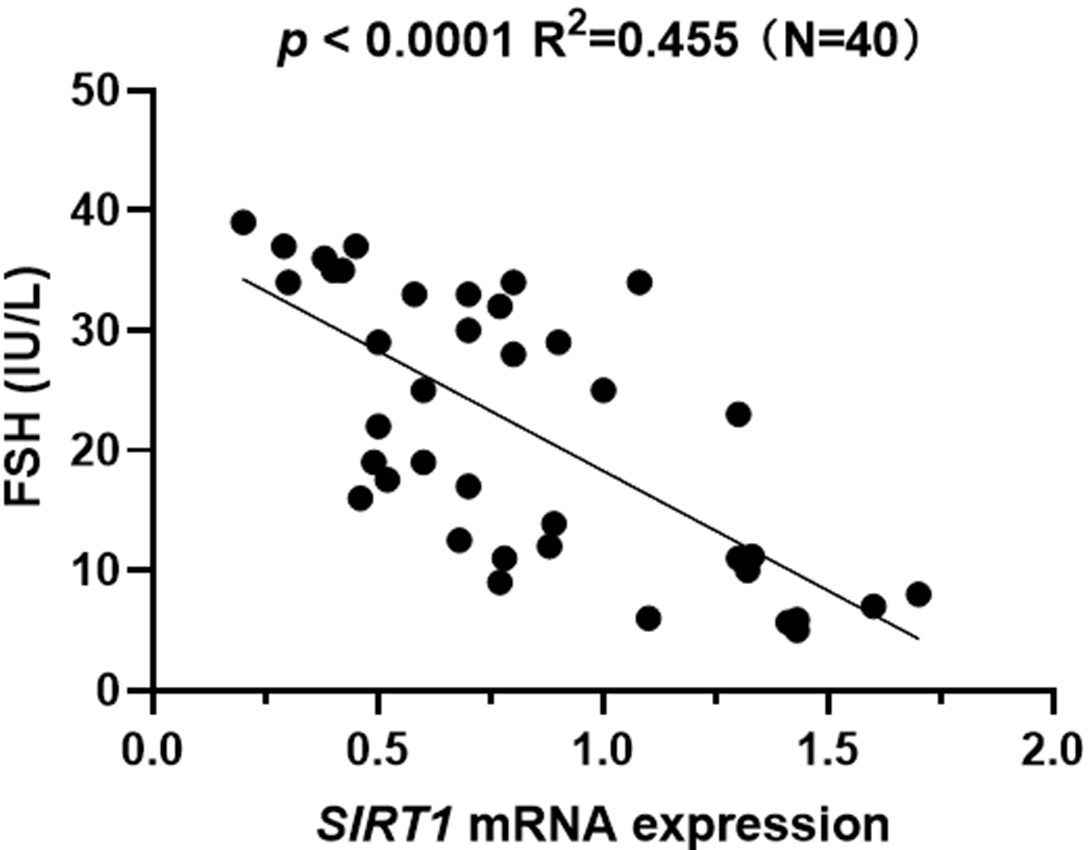

Supplement: lnag022_Supplementary_Data [file lnag022_supplementary_data.zip › SFig. S1.tif]
